# Microbiome‐Immune Interplay in Aging Brains: A Pilot Investigation from the MiaGB Cohort

**DOI:** 10.1002/alz.090734

**Published:** 2025-01-09

**Authors:** Santosh K Prajapati, Vivek Kumar, Rohit Shukla, Charles Szekeres, Yonggang Ma, Shalini Jain, Hariom Yadav

**Affiliations:** ^1^ University of South Florida, Tampa, FL USA

## Abstract

**Background:**

The microbiota‐immune‐brain axis is implicated in Alzheimer’s disease. Alterations in gut microbiota and immune functions in mild cognitive impairment (MCI) are inconsistent and remain to be understood. This study aims to investigate immune cell phenotyping and its link with gut microbial composition associated with cognitive function.

**Method:**

In this pilot study, we used data and samples from MCI (n=6) and cognitively healthy (n=22) older adults (≥60 years of age) from a large, multi‐site, community‐based prospective clinical study called MiaGB (Microbiome in Aging Gut and Brain) consortium. We recorded measures of cognitive function (using the MoCA score), immunophenotyping (using flow cytometry), and stool microbiome (using whole‐genome metagenomics and bioinformatics pipelines). Differences in immune cell populations and the microbiome were found in the two groups and correlated with cognitive function.

**Result:**

The abundance of immune cells such as granulocytes, neutrophils, T‐cells (CD4, CD8, and NK T‐cells), B‐cells, NK cells, and total monocytes were not significantly distinct. However, the levels of neutrophils and classical monocytes increased, while CD8+ T cells decreased in trend in the blood of MCI participants compared to controls. These immune cells were positively correlated with increased detrimental microbial species while negatively correlated with beneficial microbes in the MCI gut. Altered immune and microbial species discriminated MCI patients well from controls and were associated with MoCA scores.

**Conclusion:**

This study provides insights into the role of gut microbiota‐immune interactions in the process of cognitive decline in older adults and indicates the potential to be used as biomarkers to diagnose the risk of cognitive decline‐ a debilitating public health problem in older adults.

